# Angiotensin II-induced hypertension in rats is only transiently accompanied by lower renal oxygenation

**DOI:** 10.1038/s41598-018-34211-2

**Published:** 2018-11-05

**Authors:** Tonja. W. Emans, Daniela Patinha, Jaap. A. Joles, Maarten. P. Koeners, Ben. J. Janssen, C. T. Paul Krediet

**Affiliations:** 1 Internal Medicine-Nephrology, Amsterdam UMC/Academic Medical Centre at the University of Amsterdam, Amsterdam, Netherlands; 2 Nephrology and Hypertension, University Medical Centre Utrecht, Utrecht, Netherlands; 3 Institute of Biomedical and Clinical Science, University of Exeter Medical School, Exeter, United Kingdom; 4 Pharmacology and Toxicology, Maastricht University, Maastricht, Netherlands

## Abstract

Activation of the renin-angiotensin system may initiate chronic kidney disease. We hypothesised that renal hypoxia is a consequence of hemodynamic changes induced by angiotensin II and occurs prior to development of severe renal damage. Male Sprague-Dawley rats were infused continuously with angiotensin II (350 ng/kg/min) for 8 days. Mean arterial pressure (n = 5), cortical (n = 6) and medullary (n = 7) oxygenation (pO_2_) were continuously recorded by telemetry and renal tissue injury was scored. Angiotensin II increased arterial pressure gradually to 150 ± 18 mmHg. This was associated with transient reduction of oxygen levels in renal cortex (by 18 ± 2%) and medulla (by 17 ± 6%) at 10 ± 2 and 6 ± 1 hours, respectively after starting infusion. Thereafter oxygen levels normalised to pre-infusion levels and were maintained during the remainder of the infusion period. In rats receiving angiotensin II, adding losartan to drinking water (300 mg/L) only induced transient increase in renal oxygenation, despite normalisation of arterial pressure. In rats, renal hypoxia is only a transient phenomenon during initiation of angiotensin II-induced hypertension.

## Introduction

Disturbed renal oxygenation has been hypothesised as an aggravating factor during chronic kidney disease (CKD), by activation of local intrarenal pro-inflammatory and pro-fibrotic processes^1,2^. According to the hypoxia in CKD hypothesis as originally coined by Fine *et al*., hypoxia occurs before the establishment of renal histological damage. This suggests that, although there are several causes to renal damage, hypoxia is a common denominator in development of renal damage and may therefore be a new target for treatment. Hypoxia occurs when oxygen delivery and consumption are unbalanced. Glomerular hyperfiltration and subsequent increased tubular solute reabsorption increases oxygen consumption relative to the blood flow to the nephron. This cascade leads to hypoxia in the tubulo-interstitial compartment and should be detectable at an early stage of disease^3^.

In spontaneously hypertensive rats, reduced renal oxygenation was measured by ultra-microelectrodes^4^. In a remnant kidney model renal cortical hypoxia was identified by pimonidazole staining after 4 days^5^. In a progressive model of rat glomerulonephritis, wherein uninephrectomy was combined with repeated anti-Thy-1 antibody injections, hypoxia occurred from the first week on^6^. These findings imply that hypoxia indeed occurs early after initiation of mild renal damage and contributes to disease progression. Experimental reduction in oxygen delivery in isolated perfused rat kidneys exacerbates renal damage^7^, and an increase in renal oxygen consumption by administration of dinitrophenol caused proteinuria and infiltration of inflammatory cells^8^. In humans, reduced oxygenation levels were correlated to CKD severity^9^. However, so far, evidence of hypoxia in kidney disease has been based on indirect measurements or under anesthesia at a single time point. Direct tissue oxygenation (pO_2_) measurements determining hypoxia before the onset of chronic renal disease are lacking. In humans, renal oxygenation measurements are only possible by indirect measurements, such as blood-oxygen-level dependent (BOLD) magnetic resonance imaging^10–12^. Therefore, it is difficult to draw conclusions regarding the effect of hypoxia in time. Deciphering a potential role of tissue hypoxia in the initiation of renal disease asks for measurement techniques that are not restricted in time and can be done in the absence of anesthesia, which impair renal hemodynamics^13^. Renal function needs to recover from minor implantation surgery as well^13^. Therefore, in the present study we used an oxygen telemetry system to record kidney oxygenation 1) continuously 2) in awake animals throughout the experiment, and 3) after at least one week of recovery from implantation^14,15^.

A proposed link between hypoxia and initial renal changes within kidney disease is activation of the Renin-Angiotensin-System (RAS). Administration of an angiotensin-converting-enzyme (ACE) inhibitor or angiotensin receptor blocker (ARB) increased cortical oxygenation, measured by protoporphyrin phosphorescence, in anesthetised healthy rats^16^. In 5/6 nephrectomised rats, ARB administration attenuated hypoxia, assessed by pimonidazole staining^5^. These observations suggest that RAS activation is associated with early phase oxygenation changes. The RAS is a potent regulator of intrarenal hemodynamics and its activation is an acknowledged pathophysiological factor in the progression of kidney disease in humans^17^ and rats^18^. In rats, angiotensin II (AngII) infusion causes systemic hypertension, increases glomerular capillary pressure (and increases filtration fraction), and reduces renal blood flow (RBF)^19^. While the magnitude of these effects are dependent on the genetic background^20^ AngII-induced hypertensive nephrosclerosis, renal injury typically develops over time^17^, and usually becomes evident after about 7 days^21^. Previously, we observed that AngII infusion acutely decreased renal cortical oxygenation in rats^15^. That study was not designed to test for long-term consequences of AngII infusion.

In the present study, we used chronic AngII infusion to identify hypoxia before the establishment of renal damage. Oxygenation is different in renal cortex and medulla, due to structural and functional differences in relation to oxygen delivery, consumption and shunting^22^. Besides, cortical and medullary oxygenation responded differently to acute AngII infusions in awake sheep^23^. Therefore, we measured pO_2_ in both cortex and medulla. We hypothesised that renal hypoxia develops in cortex and medulla in AngII-induced hypertension prior to glomerulosclerosis and that this would be reversible by AngII type 1 (AT_1_) receptor blockade. In addition we analyzed the diurnal fluctuations in pO_2_ during AngII infusion. Under physiological conditions, we observed circadian rhythmicity in rat renal cortex and medulla^24^. Intrarenal RAS and mean arterial pressure (MAP) have circadian rhythmicity, which is associated with renal damage when disturbed^25^. To our knowledge, the effects of AngII infusion on circadian rhythm of renal oxygenation are unknown.

## Methods

### Animals

Male Sprague-Dawley rats (Charles River), weighing 300 to 350 g were co-housed in pairs. Animals were kept on a 12 h light/dark cycle with lights-on at 6am, and lights-off at 6 pm and allowed access to water and standard rat chow *ad libitum*. Temperature and humidity in the room were controlled at 21 degrees Celsius and 45%, respectively. All procedures were approved by the Animal Ethics Committee of Utrecht (DEC nr. 2014.II.03.015) and were in accordance with the Dutch Codes of Practice for the Care and Use of Animals for Scientific Purposes or with the United Kingdom Animals (Scientific Procedures) Act 1986 and associated guidelines, under licence to the Home Office.

### Surgical procedures for chronic kidney pO_2_ and MAP measurements

Oxygen sensitive carbon paste electrodes were attached to telemeters (TR57Y, Kaha Sciences, New Zealand) and calibrated for linearity before implantation^14,26^. In short, anaesthesia was induced with 5% isoflurane and maintained during spontaneous breathing of isoflurane at 2–3% added to 100% O_2_. The right kidney was exposed by laparotomy and electrodes (with telemeters in off mode) were implanted as described in detail^26^. The tip of the electrodes was placed in the cortex or medulla, at appropriate depths (0.8–1.2 or 3.5–4.0 mm, respectively). Implantation depth was verified at the end of protocol. The body of telemeter was secured on the inner abdominal muscle. Rats were kept on a warm pad separately overnight. After 24 h of recovery, the animal cage was placed on a Smartpad (TR181, Kaha Sciences) to start recording of renal tissue pO_2_.

In other rats, MAP was recorded by telemetry (TRM54P, Kaha Sciences) by placing the pressure tip in the abdominal aorta. The abdominal aorta was exposed by laparotomy using similar anaesthetic procedures. Pressure catheters were inserted in the abdominal aorta through a small hole made just above the bifurcation. After 24 h of recovery, the animal cage was placed on an external receiver pad (TR181, Kaha Sciences) to start recording of MAP. Since this is an invasive technique and may be associated with impaired renal function due to the implantation and procedures per se, a considerable period of recovery was allowed. In rats instrumented with oxygen telemeters systolic blood pressure (SBP) was measured by tail-cuff plethysmography.

### Chronic AngII infusion and AT1-blockade

Baseline MAP and pO_2_ levels were recorded for at least 5 days before AngII was administered. Osmotic pumps (Model 1002, Alzet, estimated flowrate 0.25 µl/h for 8 days) were filled with an AngII solution (30 mg/ml) in sterile NaCl with 0.01 N AcOH. The osmotic minipumps were implanted subcutaneously, under short (max 5 min) isoflurane anaesthesia between 11–12 a.m. The infusion of AngII at a dose of 350 ng/kg/min has been repeatedly shown to induce hypertension^27–29^. In our experience, in this particular rat strain, higher dosing of AngII results in cerebral haemorrhage. At the end of the AngII infusion, tail-cuff plethysmography was used to verify the magnitude of high blood pressure in the rats that were instrumented with pO_2_ telemeters.

Thirteen rats were instrumented with pO_2_ telemeters (cortex n = 6, medulla n = 7) and underwent AngII infusions as described above. In addition, in second set of 16 animals (cortex n = 5, medulla n = 6, MAP n = 5), the AT_1_ receptor blocker losartan (300 mg/L) was added to the drinking water after 6 days of AngII infusion to measure the effect of blood pressure normalisation on pO_2_. The duration of losartan treatment was 48 hours.

### Chronic AngII infusion and hydralazine

In a third set of experiments, rats were instrumented with blood pressure (n = 3) or pO_2_ telemeters (medulla, n = 3), and the peripheral vasodilator hydralazine was added to the drinking water and/or subcutaneously injected to give a dose of 5–25 mg/kg/day. Hydralazine was given either for 24 hours during AngII infusion to confirm blood pressure normalisation, or started one day before AngII infusion to investigate any blood pressure independent effects of AngII on medullar pO_2_.

### Terminal experiments

Renal function was assessed in rats (n = 18) after 8 days of AngII infusion as described previously^30^ and compared with age-matched controls (n = 8). In short, rats were anesthetised with 60 mg/kg ip pentobarbital sodium and artificially ventilated (UNO, Zevenaar, the Netherlands). Plasma was collected from the femoral artery and urine was collected from the bladder, during infusion of inulin and para-aminohippuric acid, to calculate glomerular filtration rate (GFR) and renal plasma flow (RPF), respectively. During this experiment MAP was measured via a fluid filled catheter inserted via the femoral artery. Sodium contents were measured by flame photometry to obtain tubular sodium reabsorption (TNa) and fractional TNa (FTNa) was calculated. Urine samples were also used for assessment of proteinuria. At the end of the renal function measurements study, kidneys, heart, and thoracic aorta were collected and weighted.

### Histology

Right kidneys were fixed for 24 hours in 10% formalin and embedded in paraffin. Slides (3 µm) were stained with periodic acid Schiff (PAS). These kidney sections were scored for glomerulosclerosis and tubulo-interstitial damage. Of every section fifty glomeruli were divided in five groups of severity (0–4). The percentage of glomeruli with a severity score between 2–4 are depicted in Table 1. Tubulo-interstitial damage was analyzed for infiltrate, fibrosis and atrophy. Per section twenty areas of tubulo-interstitium were given a score between 0 and 5. Mean values are depicted in Table 1. The AngII group was composed of 8 rats with proteinuria closest to the median value of the entire AngII group (n = 18). Analysis was performed by an experienced technician in a blinded manner.

**Table 1:** Renal hemodynamics (under pentobarbital anesthesia) and renal injury scores after 8 days of angiotensin II (AngII) infusion in rats.

	Control	AngII	P
**N**	**8**	**18**	
Final body weight (g)	413 ± 14	408 ± 9	NS
MAP (mmHg)	110 ± 6	134 ± 5	<0.05
GFR (µl/min/100 g)	753 ± 42	513 ± 37	<0.01
RPF (µl/min/100 g)	2492 ± 183	1961 ± 121	<0.05
Hematocrit (%)	46 ± 1	48 ± 1	NS
RVR (MAP/RBF/100 g)	25 ± 1	36 ± 4	<0.05
Filtration Fraction	0.31 ± 0.01	0.26 ± 0.01	<0.05
TNa (µmol/min)	446 ± 26	307 ± 41	<0.01
FTNa (%)	0.59 ± 0.01	0.59 ± 0.02	NS
Thoracic aorta weight (mg/100 g bw)	10.7 ± 0.3	17.0 ± 0.7	<0.001
Proteinuria (mg/day)	21 ± 3	43 ± 7	<0.01
**N**	**8**	**8**	
Glomerulosclerosis (%)	6 ± 2	10 ± 3	NS
Infiltrate (tubule)	0.11 ± 0.03	0.11 ± 0.04	NS
Fibrosis (tubule)	0.10 ± 0.02	0.30 ± 0.04	<0.001
Atrophy (tubule)	0.03 ± 0.01	0.17 ± 0.04	<0.01

MAP: mean arterial pressure, GFR: glomerular filtration rate, RPF: renal plasma flow, RVR: renal vascular resistance, RBF: renal blood flow, TNa: tubular sodium reabsorption, FTNa: fractional tubular sodium reabsorption. Data are expressed as mean ± SEM. An unpaired Student’s t-test was performed to determine p-values.

### Data analysis

**Figure 1:**
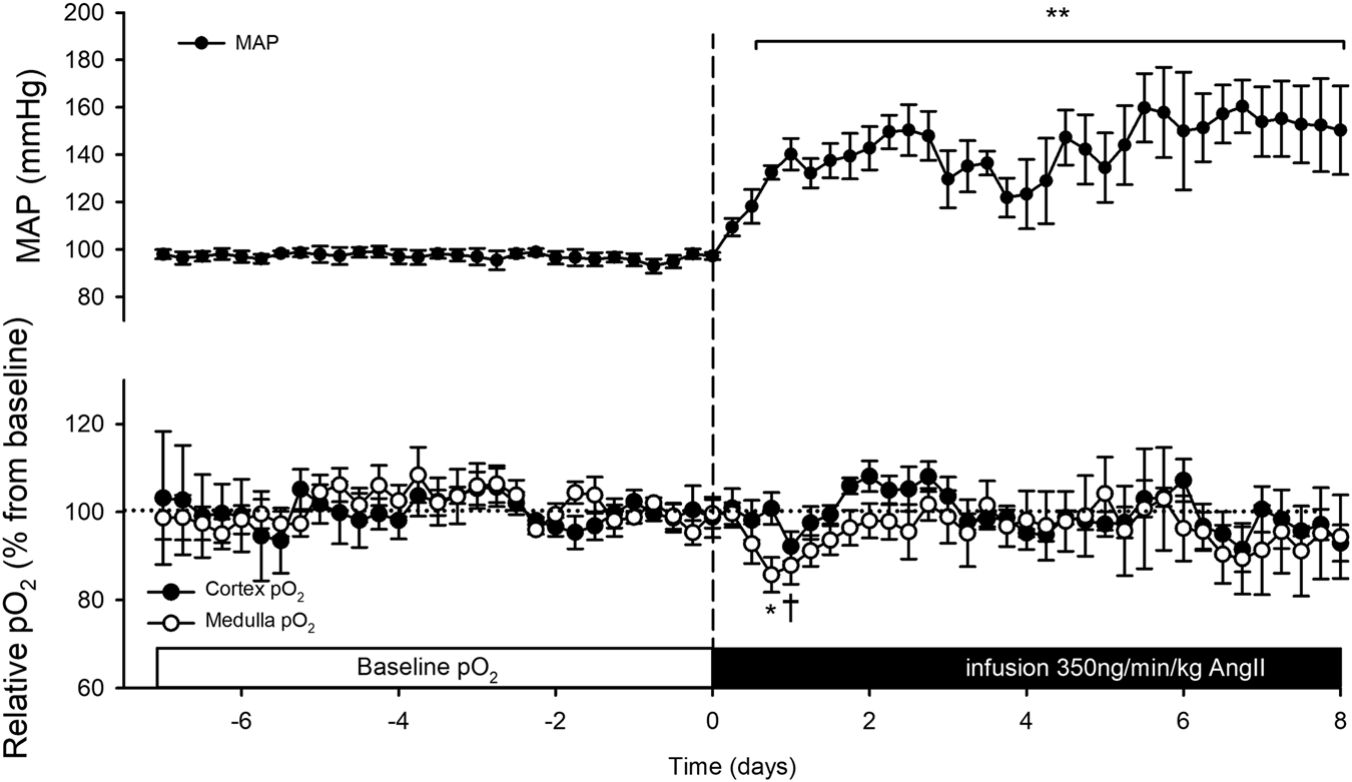
Cortical and medullary oxygenation (pO_2_) and mean arterial pressure (MAP) during 8 days of angiotensin II (AngII) infusion. Osmotic minipumps were implanted to start 350 ng/kg/min AngII infusion (dashed line). Telemetric recordings of cortical (closed circles, n = 6) and medullary (open circles, n = 7) pO_2_ were recorded continuously. Values are expressed as a percentage of the baseline period before AngII infusion. MAP was determined by telemetry (dots, n = 5) in another subset of animals. Data is presented as mean of 6 h averages ± SEM. *p ≤ 0.05 in medulla, ^†^p ≤ 0.05 in cortex, **p ≤ 0.01, all vs. baseline.

**Figure 2:**
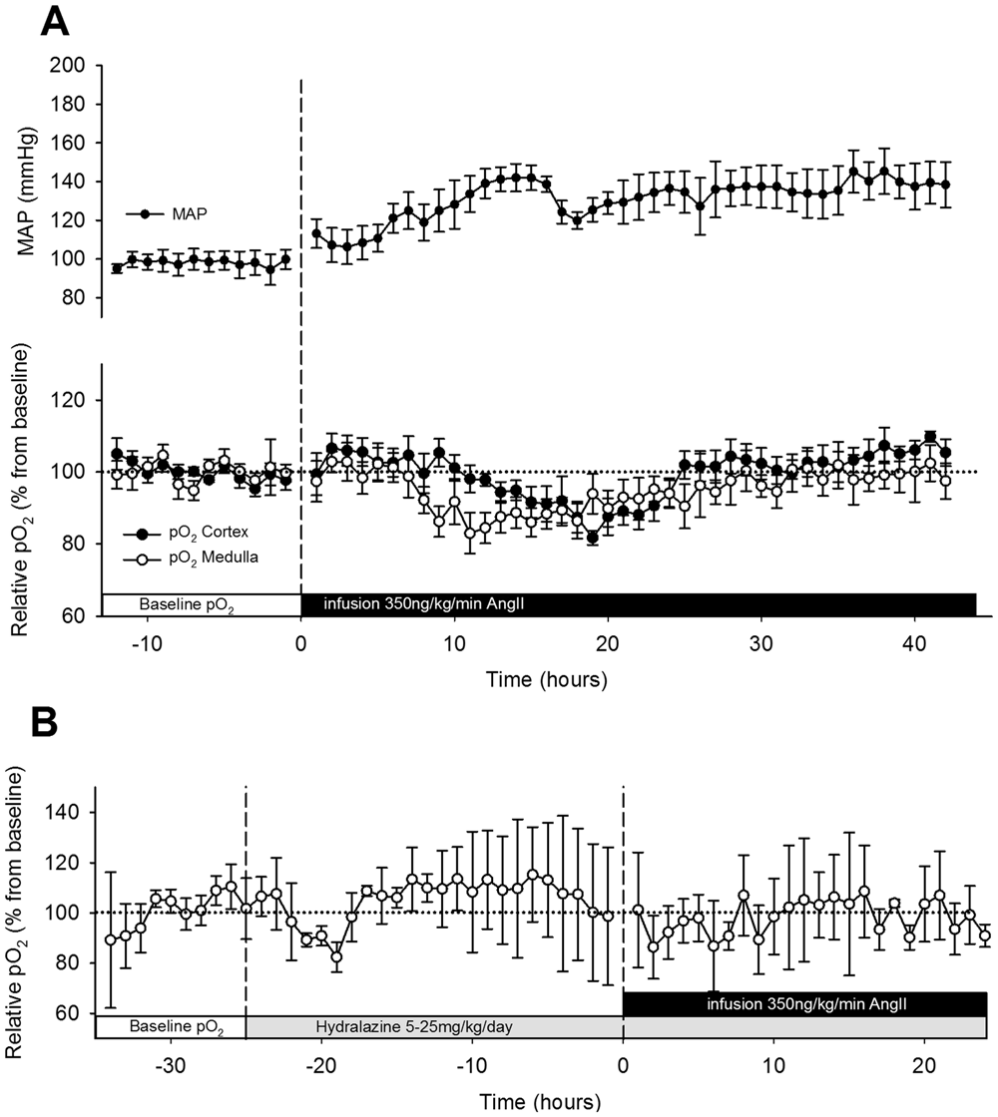
Chronic angiotensin II (AngII) infusion and hydralazine. (**A**) Cortical (n = 6, closed circles) and medullary (n = 7, open circles) oxygenation (pO_2_) and mean arterial pressure (MAP) (n = 5, dots) during the early phase of AngII infusion. Osmotic minipumps were implanted to start 350 ng/kg/min AngII infusion (dashed line). Values are expressed as a percentage of the baseline period before AngII infusion. Data is presented as mean of 1 h averages ± SEM. (**B**) Medullary (n = 3) pO_2_ during hydralazine administration (5–25 mg/kg/day) only or in combination with AngII infusion (350 ng/kg/min). Data is presented as mean of 1 h averages ± SD. Telemetric recordings were recorded continuously. (**A**,**B**) were derived from different subsets of animals.

Original pO_2_ data were recorded in nA by carbon paste electrodes attached to oxygen telemeters at 4 Hz. The off-set value was determined *post-mortem*, and subtracted from the original pO_2_ data. Baseline (nA) values were determined during 5 days before AngII infusion and set at 100% (Figs 1 and 2). Artefacts were removed when the 1st order derivative exceeded a threshold of 5 nA/s, as described^15^. Telemetry data over 1-h (AngII + losartan) and 6-h (AngII) periods are expressed as averages ± SEM. On longitudinal pO_2_ data one-way ANOVA for repeated measures was performed followed by a Tukey test. To specify the relation between MAP and pO_2_, the variations in MAP with those occurring in pO_2_ during baseline and AngII infusion were compared. This was done by pairing the mean pO_2_ and the mean MAP data in matched periods of 15 min. during a baseline period (mean from day −4 to −1) and during AngII infusion (day 3 to 6). Slopes of correlation were compared with analysis of covariance (ANCOVA). Circadian rhythmicity of MAP and renal pO_2_ was quantified as described^24^. The fitted cosine was quantified by the following parameters; mesor (circadian rhythm-adjusted mean), amplitude (half the difference between peak and nadir) and acrophase (peaktime). These parameters are shown as median with confidence interval (Table 2). Time is expressed as Zeitgeber (ZT), wherein start of lights on phase is at ZT0.

**Table 2:** Circadian rhythmicity of mean arterial pressure (MAP) and renal oxygenation (pO_2_) during angiotensin II (AngII) infusion.

	MESOR Con	MESOR AngII	Amplitude Con	Amplitude AngII	Acrophase Con (ZT h)	Acrophase AngII (ZT h)
Oxygenation Cortex (%, N = 6)	100.0 (99.3–100.7)	100.0 (99.4–100.6)	5.8 (4.7–6.8)	3.6 (2.6–4.6)*	16.9 (16.3–17.4)	18.8 (17.2–20.5)
Oxygenation Medulla (%, N = 7)	100.0 (98.8–101.2)	100.0 (98.9–101.1)	4.9 (3.6–6.3)	3.5 (2.0–4.9)	16.9 (16.0–17.7)	19.4 (17.5–21.4)
MAP (mmHg, N = 5)	95.5 (94.6–95.8)	145.4 (141.8–149.1)	1.6 (0.8–2.4)	5.5 (0.3–10.6)	18.9 (17.6–20.2)	14.1 (11.7–16.4)*

Data were analyzed by cosinor analysis (period = 24 h), lighting schedule; lights-on at 6am (ZT0), and lights-off at 6 pm (ZT12). MESOR: Circadian rhythm-adjusted mean, Acrophase: Peak time of cosine function. (median, 95% CI), *p < 0.05 vs. Control data obtained from Emans *et al*. 2017.

## Results

### AngII infusion

**Figure 3:**
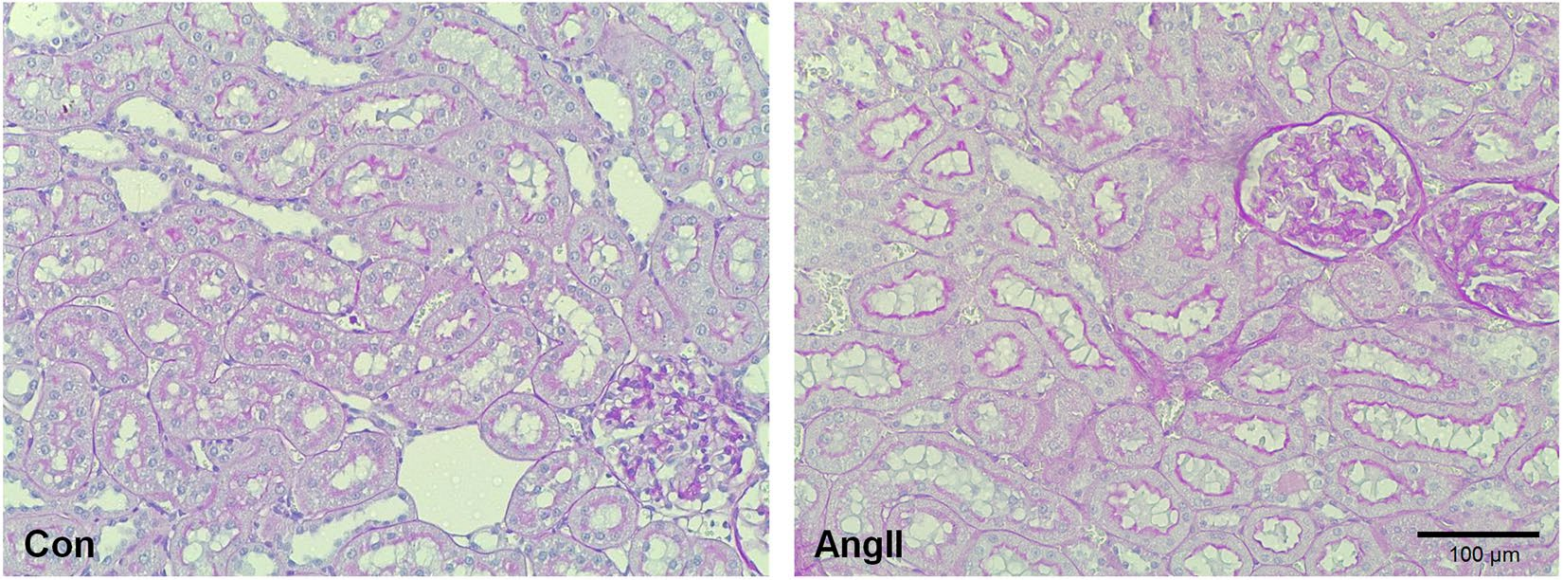
Renal morphology. Representative photomicrographs of periodic acid Schiff stained renal sections of control (Con, left) and angiotensin II-infused (AngII, right) rats. Mild fibrosis was observed in the cortex of AngII treated rats.

**Figure 4:**
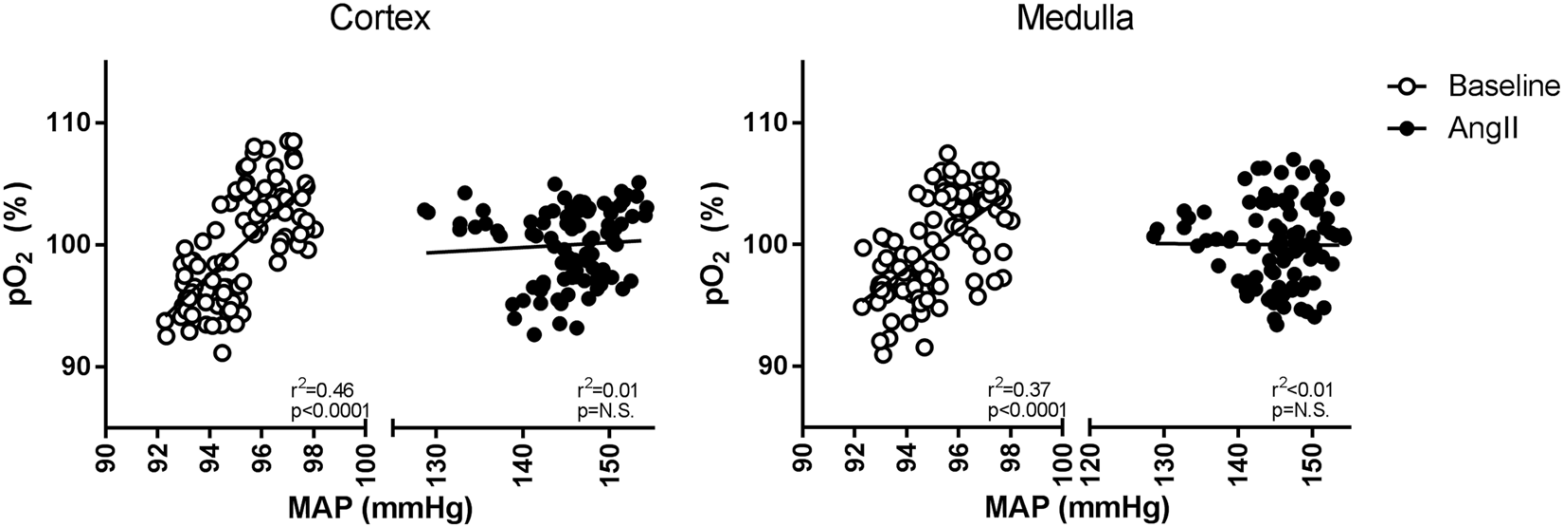
Relation between mean arterial pressure (MAP) and oxygenation (pO_2_) in cortex and medulla. In matched periods of 15 min. mean pO_2_ and mean MAP were paired during a baseline period (day -4 to -1, open circles) and during angiotensin II (AngII) infusion (day 3 to 6, closed circles). The characteristic association between MAP and pO_2_, observed during control conditions, disappeared in both cortex and medulla during AngII infusion. ANCOVA: p < 0.001 vs. baseline.

In the group instrumented with the pressure telemeter baseline MAP was 97 ± 8 mmHg. A sustained increase in MAP was observed from 6 h onwards after the start of the subcutaneous infusion of AngII. The hypertensive response was maintained throughout the full treatment period rising to maximally 150 ± 18 mmHg in rats instrumented with pressure telemeters (Fig. 1). SBP, measured in rats instrumented with oxygen telemeters, increased from 112 ± 3 to 178 ± 14 mmHg (p < 0.01). This contrasts with the transient reductions that occurred in cortical and medullary pO_2_ levels. Renal parenchymal oxygenation started to decrease after 10 ± 2 h in cortex and 6 ± 1 h in medulla (cortex vs. medulla, p = 0.05) and effects were normalised after 26 ± 3 h from the start of the infusion, in both regions (Fig. 2A). Maximal reductions in cortical and medullary pO_2_ (compared to baseline) were 18 ± 2% and 17 ± 6%, respectively. Cortical and medullary pO_2_ levels did not exceed baseline variations during the remaining AngII infusion period (Fig. 1). AngII infusion did have hemodynamic effects. GFR and RPF were reduced after 8 days in AngII infused animals compared to healthy controls (p < 0.01, p < 0.05, respectively), and TNa was proportionately reduced, so that FTNa remained constant. Proteinuria and mild renal damage were observed in AngII infused rats (Table 1 and Fig. 3). During the baseline period MAP and pO_2_ were related, in both cortex and medulla. During AngII infusion, the association between MAP and pO_2_ disappeared in the cortex and medulla (ANCOVA: p < 0.001 vs. baseline, Fig. 4).

### AT1 receptor blockade

**Figure 5:**
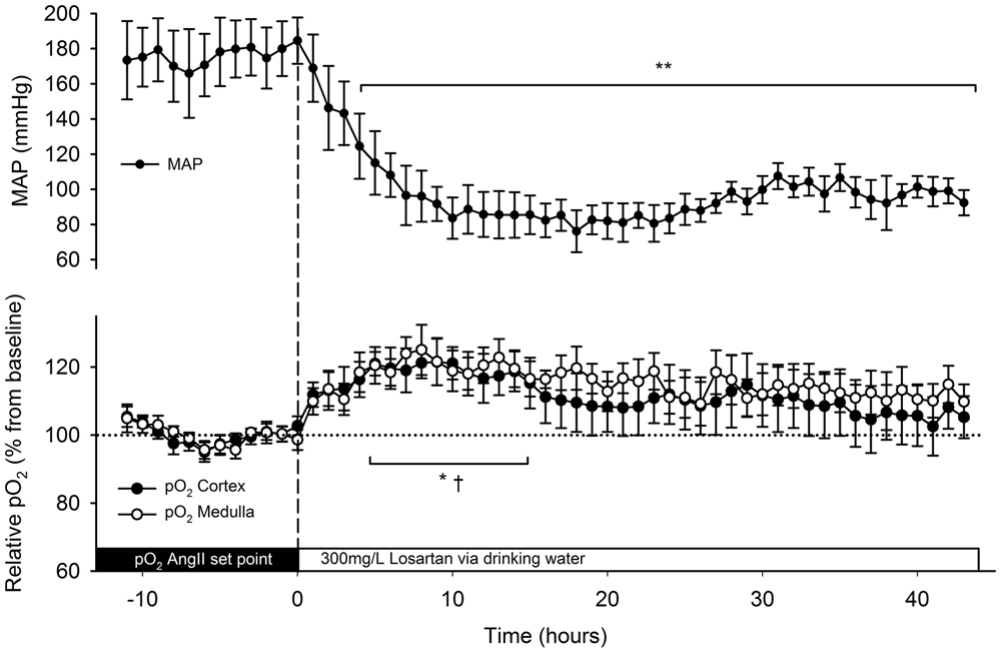
Cortical and medullary oxygenation (pO_2_) and mean arterial pressure (MAP) during angiotensin II (AngII) infusion plus oral losartan for 48 hours. Losartan was added to the drinking water (300 mg/L, dashed line). Telemetric measurements of cortical (closed circles) and medullary (open circles) pO_2_ were recorded continuously. Values are expressed as a percentage of the 12h-period before losartan. MAP was determined telemetrically (dots) in another subset of animals. Data is presented as mean of 1 h averages ± SEM. *p ≤ 0.05 in medulla, ^†^p ≤ 0.05 in cortex, **p ≤ 0.01, all vs. the average of the 12 hours prior to losartan (set point).

When losartan was added to the drinking water MAP fell to near baseline values within 12 hours (Fig. 5). Cortical and medullary pO_2_ increased to 121 ± 7% and 125 ± 7% (compared to 24 h prior to losartan, p < 0.05) during the same time frame. However, these increases in pO_2_ levels were not sustained significantly after 20 h during losartan administration, despite persistent normalised MAP.

### Chronic AngII infusion and hydralazine

Hydralazine normalised blood pressure from 122 ± 15 to 101 ± 8 mmHg at a dose of 5–25 mg/kg/day. In rats that were equipped with oxygen telemeters in the renal medulla the hydralazine infusion was started one day before the AngII infusion. The data (Fig. 2B) show that average 24 hours pO_2_ are comparable to the baseline period and also do not change during the first 24 hours of the AngII infusion (Fig. 2).

### Circadian rhythm during AngII infusion

15-min averaged data collected during day 3, 4, 5, and 6 of AngII infusion were used to calculate the acrophase, nadir, and amplitude of the circadian profile of oxygenation and MAP. In comparison to previously published control data, a significant circadian profile of MAP was intact in AngII infused rats. The MAP acrophase had shifted (from 18.9ZT to 14.1ZT). In both cortex and medulla, a significant circadian pattern pO_2_ was present in AngII infused rats with an amplitude of 3.6% [2.6, 4.6%] and 3.5% [2.0, 4.9%], respectively. However, compared to control data, cortical and medullar pO_2_ rhythms tended to phase shift, as indicated by the delayed acrophases (from 16.9ZT to 18.8 and 19.4 ZT). In the cortex, the amplitude of pO_2_ rhythmicity was slightly blunted compared to control (p < 0.05). In the medulla, a similar tendency can be observed, but this was not significant (Table 2).

## Discussion

The main new finding of this study is that renal cortical and medullary tissue oxygenation only transiently decreases during the induction of high blood pressure with AngII. Thus, in this experimental setting kidney oxygenation may be more dynamic than previously thought.

While the MAP response in our animals was comparable to values obtained in previous studies using similar doses of AngII^27–29^, the recorded changes in tissue oxygenation are surprising. Decreased oxygenation in cortex and medulla was only observed during the first 26 hours of AngII infusion. The timing of the pO_2_ nadir corresponds with the delay observed in Cyp1a1-Ren2 rats when the endogenous AngII production was activated^15^. When effects of AngII were blocked by an AT_1_ receptor antagonist cortical and medulla pO_2_ again increased only transiently, despite persistent normalisation of MAP. Together, these observations do not confirm our hypothesis that from an early point of time a reduction in renal oxygenation is the major factor involved in AngII induced renal damage. Our data rather suggest that adaptation occurs that restores pO_2_ levels. This adaptation was probably MAP independent and occurred within the first 24 hours.

The initial (first 20–30 hours) effects of AngII on oxygenation differ from those observed in the later phase (eventually leading to the development of renal injury). The declining oxygenation levels during the first 20 hours of AngII infusion are likely to be related to a temporary imbalance between oxygen consumption and supply. This data is consistent with intrarenal AngII infusion in dogs, wherein RBF decreased during the first 24 hours only^31^. This supports the idea that the initial dip in pO_2_ during AngII infusion is caused by a decrease in oxygen delivery. This is based on previous acute and longitudinal studies in awake animals, that showed that there is an immediate decrease in RBF after AngII infusion^32,33^. Because autoregulatory mechanisms relatively preserve the GFR from such a reduction, this may result in a mismatch between oxygen consumption and delivery because the filtered load is less reduced than RBF is. This phenomenon on its own causes a pO_2_ drop. At first, while the pressure effects are rather limited, AngII does limit renal blood flow, while the increased production of vasodilatory prostaglandins and nitric oxide is insufficient to overcome the flow limiting effects of AngII^31,34^. In the second phase (after the first day) the progressive rise in MAP may facilitate pressure-natriuresis by deactivation of Na^+^ channels^28^. So, at the start of AngII infusion Na^+^ reabsorption is activated, but once blood pressure rises, this is suppressed^35^. In agreement, we found TNa to be lower in the 8-day infused animals and GFR was proportionately reduced, so that FTNa remained constant. This indicates that a new homeostatic balance is accomplished between directly AngII-induced responses and compensatory hypertension-driven mechanisms. Therefore, pO_2_ levels remain constant between day 2 and 8 in this study. Renal metabolism is largely determined by Na^+^ reabsorption^36,37^. The proximal tubule is the most cost effective Na^+^ reabsorption site in terms of energy requirements^38^. Increasing Na^+^ reabsorption in less efficient segments of the tubule can potentially reduce pO_2_. Na^+^ reabsorption was reported to be enhanced in the distal nephron of mice when circulating angiotensin II is increased for 2 weeks^39^. Similarly, increased distal nephron reabsorption was reported in Cyp1a1-Ren2 rats 4 days after inducing RAS activity^40^. The potential alterations and shift of Na^+^ reabsorption to less efficient areas of the tubule do not seem to be reflected in pO_2_ at least until day 8 in our study.

An increased efficiency of oxygen use for Na^+^ reabsorption is not likely to be involved, because oxygen efficiency was found to be lowered in AngII infused animals^19,41^. All in all, our data suggest that hypoxia due to decreased delivery by AngII infusion persists in the early phase and is lost when intrarenal blood flow increases in both cortex and medulla. Indeed, according to current observations, renal oxygen levels normalised once MAP was already progressively increasing. Since our current experiment lasted only 8 days it does not preclude that renal hypoxia may return when kidney damage progresses over time. The injury response to activation of the RAS (genetic or pharmacological) depends on genetic background of the rat or mouse used. Some strains sustain almost no injury despite the same level of hypertension. This is shown for the Cyp1a1-Ren2 rat^20^ and also for the AngII infusion model^29,42^. Therefore, we need to limit our conclusions to the mild renal injury scores and proteinuria, regarding the effect of AngII on renal oxygenation.

Blockade of AngII increased cortical and medulla pO_2_ also only transiently in our animals. This suggests that oxygenation adapts within 30 hours towards hemodynamic changes induced by AT_1_ receptor signalling. Unchanged cortical pO_2_ was also found in anaesthetised rats after one week of losartan, despite a reduction in MAP^43^. When intrarenal AngII was discontinued in conscious dogs, a transient increase in RBF was observed, which suggest that increased oxygen delivery caused the pO_2_ response^31^. In CKD patients, kidney oxygenation was improved by RAS inhibitors, directly and chronically^44^. The residual oxygen increase during AT_1_ blockade could be due to the vasodilator effect of AT_2_, which is still stimulated^45,46^.

Various compensatory mechanisms could act chronically after AngII infusion (as opposed to the direct increase in nitric oxide and prostaglandins). Previously it has been demonstrated that AngII infusion stimulates the production of erythropoietin^47^. In our data, a trend towards increased haematocrit in AngII could reflect such adaptation to hypoxia. This should be subject of further study. The normalisation effects on cortical and medullary oxygenation observed in this study are probably blood pressure independent because the relation between MAP and pO_2_ was lost during AngII infusion. Nevertheless, these experiments cannot completely exclude the contribution of MAP considering that during AngII infusion it is far higher than during baseline. However, in rats made hypertensive by NOS inhibition the relation between MAP and pO_2_ was maintained in the cortex even though MAP was higher than during AngII infusion^48^. In addition, normalisation of perfusion pressure by suprarenal clamping in the 2-kidney, 1-clip rat model, did not significantly change the pO_2_ in the cortex of the unclipped kidney^49^. This perhaps suggests that normalisation of pO_2_ in our AngII-induced model is stimulus and not pressure-dependent and may be regulated by nitric oxide and AT_2_ receptors which are responsible for maintaining oxygenation in the 2-kidney, 1-clip kidneys^49^. We conclude that the initial dip of medullar pO_2_ after starting AngII is at least partially dependent on the AngII induced increase in blood pressure as this dip of medullar pO_2_ is no longer observed when AngII is given during a hydralazine regime which keeps blood pressure at baseline levels.

The impact of chronic AngII on renal oxygenation, in this study, is reflected by a blunted amplitude of cortical oxygenation compared to control data^24^ and a shift of renal (cortex and medulla) oxygenation acrophase from 2 h before the MAP peak to 5 h after the MAP peak. It is possible that the MAP rhythm could shift even more over time. In transgenic Sprague-Dawley rats that overexpress the mouse Ren-2 renin gene constitutively, the blood pressure rhythm is shifted even more and highest values occur during the first hours of the lights on period, with minimal locomotor activity. Renal hemodynamics were only mildly affected^50^. While plasma AngII levels display also a circadian rhythmicity in healthy individuals and hypertensive patients there is no correlation to blood pressure^51^.

There are some limitations to our study that merit discussion. First, considering some technical aspects, we did not perform renal blood flow measurements as combining them with intrarenal oxygenation is technically not yet feasible. For similar reasons, the recording of MAP and renal oxygenation were performed in separate animals. Future technical developments may enable combining these measurements, which is important to identify the mechanisms at stake. Ideally telemetry-based oxygenation measurements need to be delivered in absolute values; now the technique allows only a relative calibration.

Secondly, as to the generalizability of our findings, the changes in our study occurred in the absence of significant glomerulosclerosis (although we identified some tubular changes accompanying reduced GFR and proteinuria). It is plausible that when interstitial fibrosis becomes evident after longer periods of AngII infusion, i.e. after at least two weeks, renal oxygenation may well fall. Indeed, and in contrast with the current 8-day AngII infusion study, Welch *et al*. showed decreased oxygen levels in cortex and medulla in barbiturate anesthetised Wistar-Kyoto rats after 12–13 days of AngII infusion^19^. This could reflect loss of adaptation due to progressive injury, but it is also possible that anaesthesia impacts on adaptation. During first-day^52^ and prolonged^53^ infusion of AngII, Mezies *et al*. showed a disrupted oxygenation gradient, detected by BOLD MRI, that was characterised by high and low areas of pO_2_ within the rat kidney of the F344/NCrl strain. Acute pressor doses of AngII increased peritubular ischemia^54^. In humans, a 15 mmHg increase in MAP by AngII simultaneously decreased cortical oxygenation, which was attributed to decreased perfusion^11,12^. Infusion of a subpressor dose of AngII had no impact on medullar flow or pO_2_, measured by microelectrodes in anesthetised Sprague-Dawley rats^55^. That pO_2_ can be maintained along a physiological range of RBF was also shown in rabbits^56^.

In summary, the reported longitudinal measurements of cortical and medullary renal pO_2_ in conscious rats showed that AngII infusion at levels that induce hypertension do not cause sustained renal hypoxia before the onset of renal injury. Oxygenation was transiently decreased during the first day of AngII infusion, while normalisation occurred when MAP rose progressively.

## Data Availability

The data that support the findings of this study are available from the authors upon reasonable request.
